# Association Between Transient Hemodialysis and Risk of Bleeding During Peritoneal Dialysis Catheterization

**DOI:** 10.3390/jcm13237188

**Published:** 2024-11-27

**Authors:** A Young Kim, Kyu Hyang Cho, Jong Won Park, Jun Young Do, Seok Hui Kang

**Affiliations:** Division of Nephrology, Department of Internal Medicine, Yeungnam University Medical Center, Yeungnam University College of Medicine, Daegu 42415, Republic of Korea; dkdud0904@naver.com (A.Y.K.); chokh@yu.ac.kr (K.H.C.); karismatajongwon@gmail.com (J.W.P.); jydo@med.yu.ac.kr (J.Y.D.)

**Keywords:** peritoneal dialysis, chronic kidney disease, transient hemodialysis, uremic bleeding

## Abstract

**Background**: Although the risk of serious bleeding following peritoneal dialysis catheter insertion is low, pericannular bleeding can increase the risk of catheter-related infections and reduce catheter survival. We aimed to analyze the risk factors for bleeding complications during peritoneal dialysis catheter insertion and assess whether temporary preemptive hemodialysis before catheterization can reduce bleeding and improve catheter survival. **Methods**: We retrospectively analyzed bleeding complications and catheter survival in patients who underwent temporary hemodialysis prior to peritoneal dialysis catheter insertion. Cox regression analysis was performed to determine the risk factors for bleeding complications and catheter survival. **Results**: Among 336 patients, 216 and 120 comprised the non-hemodialysis and hemodialysis groups, respectively. No significant association was found between temporary hemodialysis and bleeding (hazard ratio: 1.6, 95% confidence interval: 0.87–2.95, *p* < 0.134). Multivariate analysis revealed an inverse association of platelet count (hazard ratio: 0.99, 95% confidence interval: 0.99–0.99, *p* < 0.048) and hemoglobin level (hazard ratio: 0.78, 95% confidence interval: 0.61–0.99, *p* < 0.04) with bleeding. A positive association was observed between international normalized ratio (hazard ratio: 2.24, 95% confidence interval: 1.19–4.19, *p* < 0.012) and bleeding. Conversely, temporary hemodialysis was not associated with catheter survival (hazard ratio: 1.64, 95% confidence interval: 0.63–4.25, *p* < 0.308). **Conclusions**: Temporary hemodialysis before peritoneal dialysis catheter insertion did not significantly affect bleeding risk in patients with a high risk of uremic bleeding.

## 1. Introduction

Patients with chronic kidney disease exhibit an increased bleeding tendency. Uremic bleeding may be caused by multiple factors, including platelet dysfunction due to uremic diseases, comorbidities, and medications that affect hemostatic function [1]. Fibrinogen contributes to coagulation. If the fibrinogen level is too high or too low, thrombosis or bleeding can occur, respectively [2]. In uremic coagulopathy, fibrinogen levels tend to increase, causing thrombosis rather than bleeding [3]. Although the risk of severe bleeding after peritoneal dialysis catheter (PDC) insertion is relatively low [4,5], PDC-related complications are associated with increased morbidity and mortality [5]. Pericannular bleeding may result in hematoma formation, thereby increasing the risk of catheter-related infections and affecting catheter survival [6]. Complications such as corpus luteal rupture due to coagulation dysfunction and uremic thrombocytopenia in patients with chronic kidney disease can lead to massive intra-abdominal bleeding, potentially requiring exploratory laparotomy [7]. Therefore, side effects, such as bleeding, should be considered in patients with chronic kidney disease with a high bleeding tendency.

Dialysis, erythropoietin, desmopressin, and cryoprecipitates are commonly used to prevent and treat uremic bleeding [8]. Compared with the pre-dialysis era, the incidence and severity of uremic bleeding have significantly decreased [9]. Currently, patients with advanced chronic kidney disease undergo preoperative preemptive dialysis to remove uremic substances, restore platelet function, and correct bleeding time [1,10]. However, the efficacy of dialysis in preventing and treating uremic bleeding remains uncertain [8]. Furthermore, data regarding the impact of temporary hemodialysis (HD) before PDC insertion on the risk of bleeding complications in patients at a high risk of uremic bleeding are limited. Notably, 33–63% of patients undergo incident dialysis, with the majority starting on HD [11]. Some patients demonstrating stable conditions subsequently transition to peritoneal dialysis [12]. However, data on how temporary HD affects the prognosis of these patients remain scarce. This study aimed to analyze whether temporary preemptive HD before PDC reduces the risk of bleeding in patients with chronic kidney disease with a high bleeding tendency. Additionally, we aimed to determine the risk factors associated with bleeding and catheter survival during PDC insertion.

## 2. Methods

### 2.1. Study Population

We retrospectively analyzed the medical records of all patients who underwent PDC implantation between 1 January 2012, and 30 May 2022, at a tertiary medical center in South Korea. A total of 352 patients who preferred peritoneal dialysis as first-line renal replacement therapy were selected for this study. Among them, 16 patients who underwent HD for >2 weeks or kidney transplantation before PDC insertion were excluded. None of the patients were excluded due to missing data. A total of 336 patients were enrolled in the study. This study was approved by the Institutional Review Board of Yeungnam University Hospital (2024-01-035). The requirement for informed consent was waived as the patients’ records and information were anonymized and deidentified prior to analysis.

### 2.2. Data Collection and Assessment

Data regarding age, sex, underlying comorbidities, use of antiplatelet or anticoagulant medications, bleeding status, and bleeding treatment methods were obtained. Diabetes or hypertension was defined as the use of antidiabetic or antihypertensive drugs or a history of diabetes or hypertension, respectively. Underlying comorbidities were evaluated using the age-adjusted Charlson Comorbidity Index (CCI) [13]. Blood tests, including assessment of hemoglobin, blood urea nitrogen (BUN), and creatinine (CRE) levels, were performed immediately after the patient was admitted for PDC insertion. We selected the characteristics clinically judged to be affected by catheter outcome or bleeding. Patients with specific bleeding disorders such as hemophilia were excluded from the study. The factors related to bleeding tendency included platelet count and international normalized ratio (INR), for which data could be collected retrospectively.

The patients were classified into non-HD and HD groups with and without temporary HD before PDC insertion. Notably, temporary HD was performed before PDC insertion when a high risk of uremic bleeding was identified by the attending physician based on the BUN or CRE levels or when emergency dialysis was deemed necessary. Patients who did not undergo HD before PDC insertion were defined as the non-HD group. Temporary HD was defined as HD performed within 2 weeks before PDC insertion. The femoral or internal jugular vein was used for catheterization during HD. The duration and completion of temporary HD were determined at the discretion of the attending physician. Heparin was not used for HD on the day of catheter insertion or 24 h before peritoneal dialysis surgery. Notably, PDC insertion was performed the day after admission if the physician determined that emergency dialysis was not required and the risk of uremic bleeding was low.

Bleeding events were observed for 2 weeks after PDC insertion. Bleeding was defined as any case requiring a dressing change due to oozing at the exit or incision site during the 2-week follow-up. Moderate-to-severe bleeding was defined as the need for transfusion or additional medications such as desmopressin or hemostatic agents. The reasons for catheter removal were classified into four groups: infection, malfunction, transplantation, and patient preference. Infections included PD peritonitis, exit-site infection, and tunnel infection, as defined in previous guidelines [14]. Briefly, PD peritonitis was defined as meeting two or more of the following three criteria: symptoms such as abdominal pain and/or cloudy dialysate color, a WBC count of ≥100, and a positive dialysate culture. Exit and tunnel infections were defined as the presence of redness, tenderness, or purulent drainage around the catheter. Malfunction was defined as an abnormality in infusion and/or drainage of the dialysate. Transplantation was defined as the removal of the catheter when the patient no longer required dialysis. Patient preference was defined as the removal of the catheter as the patient opted to switch from peritoneal dialysis to HD.

The patients were followed up for 1 year after PDC insertion. If the catheter was removed due to transplantation or at the patient’s request in the absence of infection or malfunction, the data were considered censored.

### 2.3. Catheter Type and Placement

Antiplatelet or anticoagulation therapy was discontinued 1 week prior to surgery in patients receiving these medications. Double-cuff, swan neck-shaped silicone peritoneal dialysis catheters were inserted in all patients by a nephrologist using a surgical or blind method with a Tenckhoff trocar. In the blind method using a Tenckhoff trocar, an incision was made under the umbilicus, and the internal cuff was affixed to the linea alba as described in a previous study [15]. Daily irrigation was performed with absolute bed rest for 1 week immediately after catheterization. In the surgical method, an incision was made in the paramedian region next to the umbilicus, and the internal cuff was affixed to the rectus muscle [15]. The patient remained on absolute bed rest on the day of catheterization, and no irrigation was performed. In both methods, PD was initiated after one week. The dressing was not changed for 1 week if no signs of bleeding were observed. Dressing was changed once daily for the remaining weeks, regardless of bleeding status.

### 2.4. Statistical Analysis

The data were analyzed using IBM SPSS Statistics for Windows (version 25.0; IBM Corp., Armonk, NY, USA). Categorical data are expressed as counts and percentages, whereas continuous data are presented as means ± standard deviation. Continuous and categorical variables were compared using *t*-tests and chi-squared tests, respectively. The differences in bleeding- or catheter-free survival rates between the two groups were compared using Kaplan–Meier survival analysis. Stratified analysis according to HD status and bleeding events was included in the Kaplan–Meier analysis. Cox regression analysis was used to analyze the risk factors for bleeding complications and catheter survival. The proportional hazard assumption was assessed using Schoenfeld residuals. All variables satisfied the assumption for bleeding as an outcome, except for the surgical method, and catheter survival as an outcome, except for the serum creatinine levels. Therefore, the surgical method was used as a stratified variable for bleeding, while serum creatinine level was excluded from the model for catheter survival. Multivariate Cox proportional hazards analysis was performed using the backward conditional method. Statistical significance was set at a *p* value of <0.05.

## 3. Results

### 3.1. Clinical Characteristics of the Patients

This study included 336 patients: 216 in the non-HD group and 120 in the HD group (Table 1). The non-HD group had older and fewer male patients compared with the HD group. In addition, the non-HD group exhibited lower CCI scores and hemoglobin levels and higher BUN and CRE levels compared with the HD group. No significant differences were observed in the prevalence of diabetes, hypertension, surgical method, use of antiplatelet or anticoagulation drugs, platelet count, or INR between the two groups.

### 3.2. Complications Following PDC Insertion

**Figure 1:**
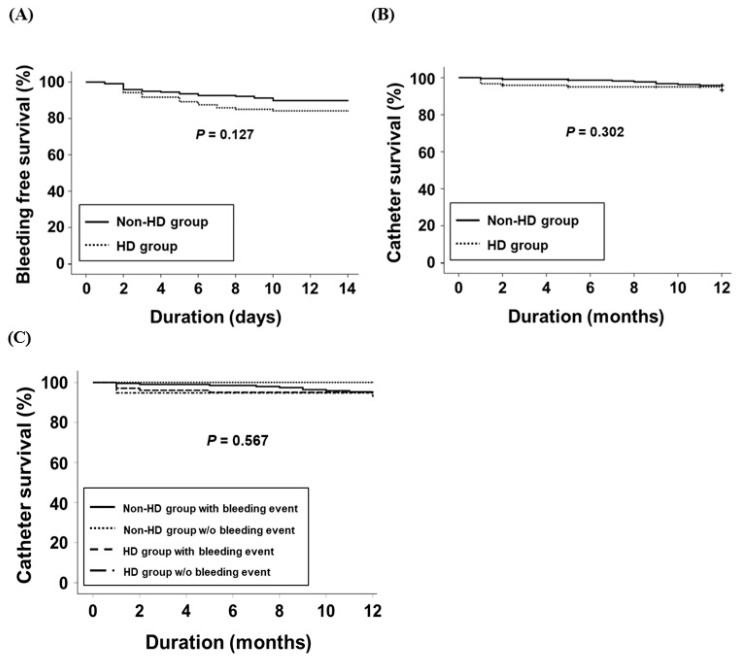
Kaplan–Meier curves for bleeding risk or catheter survival in the two groups. (**A**) Bleeding-free survival rate. (**B**) Catheter survival rate. (**C**) Kaplan–Meier curve stratified by hemodialysis status or bleeding event. The *p*-values for pairwise comparisons using log-rank tests have been included in the figure. Abbreviation: HD, hemodialysis.

A total of 22 (10.2%) and 19 (15.8%) patients in the non-HD and HD groups, respectively, developed bleeding complications (*p* = 0.130). Moderate bleeding occurred in 10 (45.5%) and 9 (47.4%) patients in the non-HD and HD groups, respectively. The bleeding-free survival rates were 89.8% and 84.2% in the non-HD and HD groups, respectively. The Kaplan–Meier analysis did not show a significant difference in the bleeding-free survival rate between the two groups (Figure 1A, *p* = 0.127).

Cox regression analysis was performed to analyze the risk factors for total bleeding. Univariate and multivariate analyses did not reveal any association between temporary HD and bleeding (Table 2). Multivariate analysis revealed a positive association between INR and bleeding.

### 3.3. Catheter Survival

The 1-year catheter survival rates were 95.8% and 93.2% in the non-HD and HD groups, respectively. Kaplan–Meier analysis did not show a significant difference in the catheter survival rate between the two groups (Figure 1B; *p* = 0.302). Additionally, the Kaplan–Meier curve stratified by HD status or bleeding events demonstrated no significant difference (Figure 1C; *p* = 0.567). Catheters were removed in 13 patients in each of the groups. The reasons for catheter removal in the non-HD group were infection in six patients (46.2%), malfunction in three patients (23.1%), kidney transplantation in two patients (15.4%), and patient preference in two patients (15.4%). In the HD group, the corresponding numbers were three (23.1%) for infection, five (38.5%) for malfunction, two (15.4%) for transplantation, and three (23.1%) for patient preference (*p* = 0.637). Cox regression analysis was performed to analyze the risk factors associated with catheter survival (Table 3). Univariate and multivariate analyses did not reveal any association between temporary HD and catheter survival. In addition, the 1-year catheter survival rates in patients with and without bleeding were 94.5% and 97.6%, respectively (*p* = 0.413).

## 4. Discussion

We analyzed the bleeding complications and catheter survival rates according to preemptive HD status before PDC in patients with chronic kidney disease. We also analyzed risk factors associated with bleeding complications and catheter survival.

When the patient characteristics of the two groups that temporarily underwent HD and those that did not were analyzed, significant differences were found in age, sex, CCI score, BUN level, CRE level, and hemoglobin level. By contrast, the non-HD group had a higher proportion of older patients, women, and patients with higher CCI scores. This may suggest that these patients were at a higher risk of requiring HD, which could explain the lower rates of consent for HD or the attending physician’s decision to forgo this procedure. The BUN and CRE levels were higher, while the hemoglobin levels were lower in the HD group compared with the non-HD group. These results may have been affected by the inclusion of many patients’ more severe renal function.

Bleeding in patients with renal failure has been well documented. Although the incidence of uremic bleeding has decreased since the introduction of dialysis, it remains a significant complication of major surgeries and invasive procedures in patients with chronic kidney disease [16]. Bleeding is a common complication, with severe bleeding occurring in 1–5% of patients undergoing peritoneal dialysis catheterization [4,17]. In our study, the overall incidence of bleeding was 12.2%, with moderate bleeding occurring in 5.7% of patients. The types of bleeding observed included bloody drainage of the peritoneal dialysis solution, pericannular bleeding around the exit site, or rectal sheath hematoma [18]. Pericannular bleeding can cause hematoma formation, which may increase the risk of catheter-related infections and decrease catheter survival [6]. Trauma, such as subcutaneous tunneling, and anticoagulant drugs are risk factors for pericannular bleeding [6]. Moreover, the use of antiplatelet and anticoagulant drugs can increase the risk of bleeding in patients with chronic kidney disease compared with the general population [19,20]. However, we did not observe a significant correlation between antiplatelet or anticoagulation drugs and bleeding. Notably, the discontinuation of these medications 1 week before surgery may have influenced our results. We used the backward conditional method to analyze bleeding risk factors. Creatinine was not included in the first model because it had insufficient statistical significance to show an independent effect on univariate analysis. However, we assumed that the model fit improved after adjusting for factors that may affect creatinine, such as hemodialysis or platelets, and creatinine was added as a meaningful variable.

Substances such as urea, guanidine succinate, parathyroid hormone, phenol, and tryptophan accumulate in patients with renal failure as clearance decreases [1]. These substances inhibit platelet function and cause defects in platelet adhesion, secretion, and aggregation, resulting in impaired hemostasis [1]. HD may improve platelet function and reduce bleeding [21,22]. However, in this study, bleeding was more frequent in the group that underwent temporary HD before PDC insertion than in the non-HD group, although the difference was not significant. The dialysis group included a higher proportion of patients with high uremic levels, which may have influenced our results.

Platelet and red blood cell counts affect bleeding time and platelet function, and anemia is a well-known risk factor for uremic bleeding [16]. However, our study did not reveal a significant association between platelet count, hemoglobin level, and bleeding. Heparin use during HD may affect platelet count. Heparin-induced thrombocytopenia type I is defined as a 30% decrease in platelet count below 100 × 10^9^ or a 50% decrease from the baseline platelet count [23]. Of the 120 patients who underwent HD, 51 received heparin. Only one patient met the criteria for heparin-induced thrombocytopenia. This finding suggests that avoiding the use of heparin or the administration of low-dose heparin during the HD period had influenced the outcomes. In addition to platelets, the key tests for assessing bleeding tendencies include activated partial thromboplastin time, prothrombin time, bleeding time, and thrombin time [24]. Prothrombin time and activated partial thromboplastin time remain normal even in patients with advanced chronic kidney disease [8]. Maintaining an INR of <1.5 during surgical treatment is considered safe [25,26]. Moreover, our study found a significant correlation between INR and bleeding. Bleeding time is the considered the most reliable test for evaluating clinical bleeding in patients with uremic disease [27]; however, these data were not collected in our study owing to its retrospective nature.

No significant association was found between bleeding and catheter-related survival. Although bleeding was a common adverse event observed in our study, severe and persistent bleeding leading to hematoma formation was extremely rare. Consequently, we did not find a direct association between bleeding and the catheter survival rate.

This study included a large number of patients, which is a notable strength. However, our study has a few limitations. First, the sample was small, and the study was conducted at a single research institute. Second, the need for patients to undergo temporary HD due to a high uremic bleeding tendency was determined by the attending physician. Subsequent randomized controlled trials focusing on similar uremic conditions would be helpful for identifying the effects of temporary HD.

## 5. Conclusions

Temporary HD before PDC insertion did not significantly affect bleeding in patients with a high risk of uremic bleeding. Our study revealed that PDC insertion must be initiated immediately when patients require dialysis, particularly those already undergoing this procedure.

## Figures and Tables

**Table 1:** Characteristics of the patients.

	Non-HD (n = 216)	HD (n = 120)	*p*-Value
Age (years)	57.5 ± 12.6	52.4 ± 14.0	<0.001
Sex (male)	125 (57.9%)	87 (72.5%)	0.008
CCI score	5.1 ± 1.9	4.7 ± 2.0	0.028
Diabetes	131 (60.6%)	70 (58.3%)	0.678
Hypertension	180 (83.3%)	93 (77.5%)	0.189
Use of AP or AC agents	70 (32.4%)	36 (30%)	0.649
Blood urea nitrogen (mg/dL)	81.7 ± 24.7	97.2 ± 32.6	<0.001
Serum creatinine (mg/dL)	7.5 ± 2.4	10.3 ± 4.2	<0.001
Platelet (K/μL)	206 ± 74	215 ± 77	0.275
Hemoglobin (g/dL)	9.3 ± 1.3	8.9 ± 1.5	0.008
INR	1.0 ± 0.1	1.0 ± 0.3	0.241
Operation method			0.592
Blind method	136 (63%)	72 (60%)	
Surgical method	80 (37%)	48 (40%)	

Data are expressed as the means ± standard deviation for continuous variables and as numbers (percentages) for categorical variables. *p*-values were calculated using a *t*-test, while Pearson’s χ^2^ test was used to analyze categorical variables.

**Table 2:** Cox regression analysis of the factors contributing to bleeding.

	Univariate		Multivariate	
	HR (95% CI)	*p*-Value	HR (95% CI)	*p*-Value
HD group (ref: non-HD group)	1.60 (0.87–2.95)	0.134	1.81 (0.93–3.52)	0.082
Age (increase by 1 year)	1.01 (0.98–1.03)	0.583	—	
Sex (ref: male)	0.79 (0.41–1.53)	0.488	—	
CCI score (increase 1 score)	1.09 (0.93–1.26)	0.285	—	
Use of AP or AC agents	1.39 (0.74–2.60)	0.308	—	
BUN (increase by 1 mg/dL)	1.00 (0.99–1.01)	0.479	—	
Serum Cr (increase by 1 mg/dL)	0.95 (0.86–1.05)	0.297	0.90 (0.80–1.00)	0.051
Platelet (K/μL)	1.00 (0.99–1.00)	0.077	0.99 (0.99–1.00)	0.052
Hemoglobin (g/dL)	0.83 (0.66–1.05)	0.114	0.79 (0.61–1.00)	0.054
INR	2.17 (1.28–3.68)	0.004	2.17 (1.20–3.92)	0.010

Multivariate analysis was adjusted for HD group, age, sex, CCI score, use of AP or AC agents, BUN levels, serum Cr levels, platelet count, hemoglobin levels, and INR, and stratified by surgical method due to the violation of the proportional hazard assumption. The analysis was performed using the backward conditional method. Abbreviations: HD, hemodialysis; CCI, Charlson Comorbidity Index; AP, antiplatelet; AC, anticoagulant; Cr, creatinine; BUN, blood urea nitrogen; INR, international normalized ratio.

**Table 3:** Cox regression analysis of factors contributing to catheter survival.

	Univariate	
	HR (95% CI)	*p*-Value
HD group (ref: non-HD group)	1.64 (0.63–4.25)	0.308
Age (increase by 1 year)	0.99 (0.96–1.04)	0.905
Sex (ref: male)	0.35 (0.10–1.23)	0.103
CCI score (increase 1 score)	1.04 (0.82–1.33)	0.734
Use of AP or AC agents	1.51 (0.57–3.96)	0.405
BUN (increase by 1 mg/dL)	1.01 (0.99–1.02)	0.364
Platelet (K/μL)	1.00 (0.99–1.01)	0.431
Hemoglobin (g/dL)	1.13 (0.81–1.60)	0.471
INR	1.18 (0.23–6.19)	0.842
Operation method (ref: blind)	0.88 (0.33–2.38)	0.803

Multivariate analysis was adjusted for HD group, age, sex, CCI score, use of AP or AC agents, BUN levels, platelet count, hemoglobin levels, INR, and surgical method. Serum Cr was excluded, as it violated the proportional hazard assumption. The analysis was performed using the backward conditional method. As no variables were included in the model, the results of the multivariate analysis are not presented. HD, hemodialysis; CCI, Charlson Comorbidity Index; AP, antiplatelet; AC, anticoagulant; BUN, blood urea nitrogen; INR, international normalized ratio.

## Data Availability

The raw data supporting the conclusions of this study will be made available by the corresponding author upon request.
